# Correction: Latent class analysis of IPOs in the Nordics

**DOI:** 10.1371/journal.pone.0293642

**Published:** 2023-10-25

**Authors:** The PLOS ONE Staff

In Table 11, the first row should not appear in bold. The publisher apologizes for the error. Please see the correct Table 11 here.

**Table 11:** Least squares regressions.

**First-day returns**	*Constant*	12.177*	12.685*	12.434*	12.741*
		(0.076)	(0.088)	(0.072)	(0.090)
	*Offer Size*		-0.420		-0.257
			(0.876)		(0.923)
	*Market Return*			1.274	1.254
				(0.503)	(0.499)
	*Observations*	314	314	314	314
	*R-square*	0.088	0.088	0.089	0.089
**First-week returns**	*Constant*	18.865**	19.088**	18.778**	19.068**
		(0.014)	(0.022)	(0.014)	(0.022)
	*Offer Size*		-0.185		-0.243
			(0.943)		(0.925)
	*Market Return*			-0.429	-0.448
				(0.813)	(0.803)
	*Observations*	314	314	314	314
	*R-square*	0.106	0.106	0.106	0.106
**Four-week returns**	*Constant*	20.301*	22.600**	19.859*	22.495**
		(0.058)	(0.050)	(0.061)	(0.048)
	*Offer Size*		-1.899		-2.205
			(0.550)		(0.493)
	*Market Return*			-2.185	-2.356
				(0.262)	(0.231)
	*Observations*	314	314	314	314
	*R-square*	0.093	0.094	0.095	0.097

Note: The dependent variable in the least squares regressions with robust standard errors is the first-day return, the first-week return respective the four-week return on the issuing firms’ securities. *Offer Size* is the logarithm of the number of shares times the offer price for those shares in the IPO, and *Market Return* is the stock market return in percent in the relevant market on the issue date of the IPO (i.e., the return on OMXC20 if the IPO’s country of origin is Denmark, the return on OMXH25 if the IPO’s country of origin is Finland, the return on OMXO20 if the IPO’s country of origin is Norway, and the return on OMXS30 if the IPO’s country of origin is Sweden). Dummy variables for year, with 2019 as the baseline category, the IPO’s country of origin, with Sweden as the baseline category, and the economic sector classification of the IPO according to the Thomson Reuters Business Classification, with the technology sector as the baseline category, are included in the regressions. *Observations* is the number of IPOs in a regression, and *R-square* is the fraction of the dependent variable that is explained by the model. *p*-values are in parentheses. Significance levels:

*p<0.1,

**p<0.05 and

***p<0.01.
