# Supplementary material for: Latent class analysis of IPOs in the Nordics
Source: PLoS One. 2021 Nov 3;16(11):e0259510. doi: 10.1371/journal.pone.0259510 (PMC8565723; doi:10.1371/journal.pone.0259510)
Supplement: S1 Appendix — (DOCX) [file pone.0259510.s002.docx]

| *Year* | *Observations* | *Min* | *Median* | *EW Mean* | *VW Mean* | *Max* | *SD* |
| --- | --- | --- | --- | --- | --- | --- | --- |
| 2009 | 1 | -80.16% | -80.16% | -80.15% | -80.15% | -80.16% | - |
| 2010 | 31 | -100.00% | -32.69% | -7.99% | 252.06% | 7.35E9% | 181.69% |
| 2011 | 15 | -99.99% | -26.39% | -67.90% | -67.53% | 1.06E4% | 65.41% |
| 2012 | 8 | -100.00% | -42.39% | -60.82% | -43.66% | 2.05E5% | 117.77% |
| 2013 | 17 | -99.52% | 0.00% | -7.83% | 17.23% | 3.24E4% | 36.25% |
| 2014 | 33 | -100.00% | 77.83% | 7.90% | 566.69% | 3.79E5% | 107.14% |
| 2015 | 50 | -100.00% | 54.23% | 133.72% | 306.34% | 1.50E7% | 117.36% |
| 2016 | 40 | -100.00% | 115.35% | 561.83% | 20.78% | 2.50E9% | 158.57% |
| 2017 | 71 | -100.00% | 129.89% | 976.98% | 224.52% | 2.24E11% | 144.75% |
| 2018 | 38 | -100.00% | 14.29% | 98.03% | 192.29% | 5.94E12% | 175.45% |
| 2019 | 10 | -70.87% | 256.36% | 889.14% | 749.29% | 8.52E6% | 89.21% |
| All | 314 | -100.00% | 18.68% | 174.84% | 188.77% | 5.94E12% | 139.32% |

**Table A.1:** Descriptive statistics of first-day returns on the issuing firms’ securities, where the mean returns and the standard deviation of returns have been transformed to the four-week horizon.

Note: *Observations* is the number of IPOs during a specific *Year*, *Min* is the minimum return, *Median* is the median return, *EW Mean* is the equally weighted mean return, *VW Mean* is the value weighted mean return, *Max* is the maximum return, and *SD* is the standard deviation of returns. The offer size of an IPO, which is the number of shares times the offer price for those shares, divided by the offer sizes of all IPOs in a given *Year* is used as the weight when calculating *VW Mean*.

| *Year* | *Observations* | *Min* | *Median* | *EW Mean* | *VW Mean* | *Max* | *SD* |
| --- | --- | --- | --- | --- | --- | --- | --- |
| 2009 | 1 | -36.35% | -36.35% | -36.35% | -36.35% | -36.35% | - |
| 2010 | 31 | -98.97% | -13.46% | -14.26% | 27.39% | 1.11E4% | 70.83% |
| 2011 | 15 | -89.05% | -4.10% | -20.74% | -27.09% | 147.99% | 34.81% |
| 2012 | 8 | -30.97% | -8.18% | 71.42% | -23.20% | 2.33E4% | 88.19% |
| 2013 | 17 | -76.57% | -1.63% | -7.80% | 2.29% | 76.30% | 21.38% |
| 2014 | 33 | -99.80% | 26.58% | 15.22% | 43.47% | 342.29% | 40.46% |
| 2015 | 50 | -99.98% | 10.04% | 14.65% | 35.04% | 440.14% | 54.38% |
| 2016 | 40 | -99.99% | 16.22% | 46.07% | -1.98% | 1.59E4% | 70.19% |
| 2017 | 71 | -94.54% | 19.71% | 83.89% | 32.11% | 8.68E4% | 88.10% |
| 2018 | 38 | -97.20% | -0.76% | 3.47% | 34.95% | 3.95E4% | 65.22% |
| 2019 | 10 | -39.13% | 18.75% | 56.84% | 47.25% | 557.33% | 44.69% |
| All | 314 | -99.99% | 4.10% | 26.17% | 23.09% | 8.68E4% | 67.02% |

**Table A.2:** Descriptive statistics of first-week returns on the issuing firms’ securities, where the mean returns and the standard deviation of returns have been transformed to the four-week horizon.

Note: *Observations* is the number of IPOs during a specific *Year*, *Min* is the minimum return, *Median* is the median return, *EW Mean* is the equally weighted mean return, *VW Mean* is the value weighted mean return, *Max* is the maximum return, and *SD* is the standard deviation of returns. The offer size of an IPO, which is the number of shares times the offer price for those shares, divided by the offer sizes of all IPOs in a given *Year* is used as the weight when calculating *VW Mean*.
