# Supplementary material for: Latent class analysis of IPOs in the Nordics
Source: PLoS One. 2021 Nov 3;16(11):e0259510. doi: 10.1371/journal.pone.0259510 (PMC8565723; doi:10.1371/journal.pone.0259510)
Supplement: Attachment — Submitted filename: Appeal (PONE-D-21-08567R1).pdf [file pone.0259510.s005.pdf]

Appeal on the decision to reject the paper "Latent class analysis of IPOs in the Nordics" (PONE-D-21-08567R1) co-authored by Mikael Bask and Anton Läck Nätter

In his decision letter, professor J. E. Trinidad Segovia (Academic Editor) write that the *"authors have not been able to provide a convincing answer to some of the major concerns, such us, what is the contribution of this research to the existing literature on this topic"*.

Are we not explaining convincingly in the paper what the contribution of our research is to the existing literature? Yes, we do that. For example, let us cite rows 54-61 in the introductory section of the paper:

"Latent class analysis (LCA) is used to examine the relationship between the offer size of an IPO, the market return on the IPO's issue date and the pricing of the IPO. LCA is useful when it is suspected that groups of IPOs exist in the sample with different properties but it is not easy to identify those groups [10]. LCA identifies the groups—or latent classes—and helps us to understand their properties and how likely it is that an IPO belongs to a certain class. Specifically, LCA aims to identify latent classes of IPOs that share common traits and treats the sample as heterogeneous regarding the relationships between the involved variables. This means that the empirical analysis herein is not based on a theoretical IPO model derived from economic principles. To the best of our knowledge, this is the first study on the pricing of IPOs using LCA."

In the last sentence (in blue), we write that we are not aware of any other study of IPOs that uses latent class analysis (LCA). We also write in the same paragraph what LCA does with IPO data. That is, *"LCA identifies … latent classes [of IPOs that share common traits] … and helps us to understand their properties and how likely it is that an IPO belongs to a certain class"* (quotes from the blue-colored text).

Let us also cite rows 192-199 in the discussion section of the paper:

"What is the value-added of using LCA when studying IPOs? LCA treats the sample with IPOs as heterogeneous regarding the relationships between the involved variables. This should be contrasted with ordinary regression analysis, which assumes that the dependent and explanatory variables behave uniformly over the whole sample. In other words, LCA is able to identify more than one group—or latent class—of IPOs that share common traits. For example, LCA revealed in this study that there are four latent classes of IPOs in the Nordics with different qualitative properties. Hence, LCA is able to detect patterns in a sample that ordinary regression analysis might miss. For this reason, LCA is a valuable complement to traditional regression analysis when studying IPOs."

In the paragraph (in blue), we explain what the value-added is of using LCA when studying IPOs. That is, that LCA is able to detect patterns in the dataset that traditional regression analysis might miss and that LCA, for this reason, is a valuable complement to traditional regression analysis. Recall that we are not aware of any other study that uses LCA when studying IPOs (see rows 60-61).

Taken together, we convincingly explain in the paper what the contribution of our research is to the existing literature. However, we are not only doing this in the paper, we also write the following in the letter to professor J. E. Trinidad Segovia when re-submitting our paper to PLOS ONE:

"Our main contribution to the literature is our use of latent class analysis (LCA) as the statistical tool when analyzing IPO data. LCA is extensively used in psychology because it takes a person-centered approach to the data. In the same manner, LCA is a useful tool for analyzing IPO data because of its IPO-centered approach. As explained in the paper, LCA identifies latent classes of IPOs that share common traits and also treats the sample as *heterogeneous* in the relationships between the involved variables. This is in contrast with ordinary regression analysis, which assumes that all the variables behave

uniformly over the whole sample. … To the best of our knowledge, our study is the first on the pricing of IPOs using LCA" (italics in original).

Notice the phrases *"our main contribution to the literature"* and *"as explained in the paper"* in the quoted text (in blue).

In summary, it is not correct that we are not explaining convincingly in the paper what the contribution of our research is to the existing literature is.

What is the reviewer of our paper writing about our paper?

"Although the aimed contribution of the manuscript is now more clear, it also opens up the issue of the significant contribution of the paper. Just applying another methodology to a well-researched topic, does not constitute a contribution in itself. Only when the new method advances our understanding of the field, this warrants publication. I still fail to see the contribution of the manuscript".

The reviewer is not convinced that our paper contains a *"significant contribution"*. This question is not relevant for publication in PLOS ONE as long as the research is performed in a rigorous manner. Let me cite what professor Emily Chenette, the Editor-in-Chief, wrote in her blog post on August 12th this year: PLOS ONE *"was the very first multidisciplinary journal that aimed to publish all ethically and methodologically rigorous research, regardless of the novelty of the findings or the perceived impact of the work"*. (Let us just say in passing that this was the reason why we submitted our paper to the journal.)

The reviewer also argues that he/she fails to see what the contribution of the paper is (see the comment *"I fail to see the contribution of LCA to the IPO literature"*), but he/she also writes in the same paragraph that *"the aimed contribution of the manuscript is now more clear"*. Hence, this is a similar comment, but in other words, as what professor J. E. Trinidad Segovia write in his decision letter. For this reason, we refer to what we write above in our response to his claim.

The reviewer's comments, one by one:

1. The meta study that the reviewer refers to is Engelen et al. [7] that uses a sample of 123 empirical studies on IPOs. None of the studies in the meta study use LCA (and none of the studies use Nordic IPO data, which is acknowledged by the reviewer). Moreover, the reviewer asks us what LCA teaches us regarding IPOs that we did not know before. In the second and third paragraphs in the paper's discussion section (i.e., rows 185-199, where rows 192-199 are quoted above), we not only write what the value-added is with LCA when studying IPOs, we also describe the latent classes found in the IPO data when using LCA.
2. The reviewer asks us in what way Nordic IPO data can advance our understanding on IPO underpricing. However, directly after having asked us this question, the reviewer refers to his/her first comment that we already have answered (see comment #1).
3. Correct, the descriptive statistics of the dataset do not contain any new insights. The reviewer seemed to believe, after having read the first version of our paper, that our main contribution of the paper was the descriptive statistics of the dataset and not the LCA of the dataset. Actually, it was because of this reviewer that we decided to include the phrase "latent class analysis" in the title of the paper. It should be noted that it is standard in the literature to present descriptive statistics of a dataset before analyzing it.
4. Nothing to comment on.
5. The reviewer asks us whether the regulatory frameworks in Denmark, Finland, Norway and Sweden are similar enough to justify pooled IPO data. Yes, the regulatory frameworks are similar enough (e.g., *"the legal environment for listing and trading of securities is to a large extent*

harmonised with the EU legislation" in [11], and [12] explains that the Norwegian legislation is *"subject to a number of laws and regulations (which in general incorporates the applicable EU legislation)"*). We now clarify this by revising the last sentence in the first paragraph in the section "Dataset and descriptive statistics".

The reviewer ends the report with the following concluding words: *"I fail to see the contribution of LCA to the IPO literature"*. This is the same comment as in ingress of the report (cf., *"I fail to see the contribution of LCA to the IPO literature"*). Once more, we refer to what we write above in our response to professor J. E. Trinidad Segovia's claim that the *"authors have not been able to provide a convincing answer to some of the major concerns, such us, what is the contribution of this research to the existing literature on this topic"*.

The following changes have been made in the manuscript (with track changes):

- Rows 79-81: We have revised the sentence (see our reply to comment #5 by the reviewer).
- Rows 129-130: We have updated the numbering of references.
- Row 214: We had missed an "i" in the title of the article.
- Rows 220-224: We have added two new references (that affects the numbering of references).
- Rows 225 and 227: We have updated the numbering of references.

/Mikael Bask and Anton Läck Nätter
