# Supplementary material for: Latent class analysis of IPOs in the Nordics
Source: PLoS One. 2021 Nov 3;16(11):e0259510. doi: 10.1371/journal.pone.0259510 (PMC8565723; doi:10.1371/journal.pone.0259510)
Supplement: Attachment — Submitted filename: POSE_2021_May_referee report_2.docx [file pone.0259510.s003.docx]

Referee report for manuscript PONE-D-21-08567 i

Thank you for submitting an interesting paper.

The paper is updating research on the important market for Nordic IPOs, not because of its size but because of that I has been a breeding ground for important innovation. It is also a market with relatively strict regulatory requirements on new listings in international comparison and hence can serve as a best practice ground, see Westerholm (2007). The paper by Bask and Lack Natter looks at all markets in the region which I think is a good approach as they are economically interconnected and comparable in terms of regulation, also their stock exchanges are either merged or co-listing each other’s shares. The paper finds that

I have a few suggestions how to improve the final version of the paper

**1.** line 38 whether the average underpricing, or overpricing, of IPOs is corrected after one or four weeks 38 of trading. This is a dimension in the pricing of IPOs that is under-researched.

I would propose say: “Intermediate and long-run underperformance is relatively under-researched” AND footnote some of the papers that do look at long run underperformance e.g. Pseudo Market Timing and the Long-Run Underperformance of IPOs by Paul Shultz (2003), check google scholar for later referenced to this paper. Westerholm (2007) also reports intermediate to long run returns.

**2.** Line 59 The rest of the note is organized as follows – **Should you use “paper” rather than “note” in my view this is a full paper not a note on previous work?**

**3.** The paper examines a total of 314 IPOs in Denmark (31 IPOs), Finland (43 IPOs), Norway (65 IPOs) and Sweden 63 (175 IPOs) issued by firms during the period of November 2009 through June 2019. **The number seems a little low do you only include major list companies?**

**4. Empirical analysis You compare returns and standard deviations for different horizons.**

**4.1 I suggest you need to consider a robustness check when you compute excess returns for the IPO stock corrected for a market index during the same period. Attention you do already report the market index and show they don’t move much during 1 or 4 weeks so I am happy with that, if you wanted you could compute the difference between stock returns and index returns for the respective periods.**

**4.2 You should at the very least annualise the returns so that they are comparable across different horizons. (You can annualise the 1 week standard deviation by multiplying by square root out of 52). If you prefer not to annualise as these return may look large, transform them all to one month returns and compare. This can be separate appendix table only comparing this for table 1 and 4.**

**5.** Conclusions: think of something general we can learn from your analysis with regards to market efficiency, of important to investors, or regulators.

Thank you for the opportunity to read your work and best of luck with the revision of the final version.

I do not need to see these corrections and I will recommend an acceptance of your paper.

Best wishes

Referee

REFERENCES

Westerholm, P. Joakim, 2007, Industry Clustering in Nordic Initial Public Offering Markets, International Review of Finance

<https://onlinelibrary.wiley.com/doi/abs/10.1111/j.1468-2443.2007.00058.x>

period investigated 1991-2002

Schultz, Paul, 2003, Pseudo Market Timing and the Long-Run Underperformance of IPOs, Journal of Finance

<https://onlinelibrary.wiley.com/doi/full/10.1111/1540-6261.00535?casa_token=sS3B_StF2DcAAAAA%3AdpOxc3onwxsars7vy1aQBGxhb4ZRJw8W5pzdwADgtYqRiOpIdVOj78HL3JpaMbQn5Nj748wAi7F8YA>
