# Supplementary material for: Latent class analysis of IPOs in the Nordics
Source: PLoS One. 2021 Nov 3;16(11):e0259510. doi: 10.1371/journal.pone.0259510 (PMC8565723; doi:10.1371/journal.pone.0259510)
Supplement: Attachment — Submitted filename: Letter to PLOS ONE Reviewer #3 (2nd round).docx [file pone.0259510.s004.docx]

Dear Reviewer #3,

First, we would like to thank you for taking the time to read our paper *“Market Return, Offer Size and the Underpricing of IPOs in the Nordics”* (now under the title *“Latent Class Analysis of IPOs in the Nordics”*) and for offering suggestions that will improve the quality of the paper.

Responses to your comments:

1. A recent meta study on IPO pricing [Engelen, P. J., Heugens, P., Van Essen, M., Turturea, R., & Bailey, N. (2020). The impact of stakeholders’ temporal orientaton on short-and long-term IPO outcomes: A meta-analysis. Long Range Planning, 53(2), 101853.] addresses the pricing of IPOs according to those two dimensions (proceeds and underpricing). The authors should embed their manuscript within the IPO literature and clearly demonstrate their contribution. What do we learn from the current study which was not addressed in this meta-study?

In the revised paper, in Section 1, we refer to the meta-analysis by Engelen et al. (2020). Essentially, the empirical studies included in the meta-analysis measure the time horizon in month or years, whereas the time horizon in our paper is measured in weeks. Hence, we add to the literature on intermediate-run performance in the pricing of IPOs. We now write in Section 1 that through the empirical analysis we have learned that there are four latent classes in the IPO data with different qualitative properties. Hence, there is considerable heterogeneity in the IPO data that would have been hard to discover with ordinary regression analysis.

2. In what way does the focus on 4 Nordic countries help us to better understand IPO underpricing. The mere fact that Nordic IPO markets are under-analyzed is a poor motivation for the study. There are several cross-country studies in the IPO literature, including the Nordic countries. So what make the focus on those 4 countries so particular that it helps us to develop our understanding of the IPO phenomenon?

In the revised paper, we have removed the text suggesting that there are relatively few studies on the Nordic IPO market because it is relatively small. We have also removed the text claiming that there are relatively few studies on the Nordic IPO market, although we still believe that there are few published studies in peer-reviewed international journals on this particular market. There are several unpublished studies on the Nordic IPO market. However, we have chosen to not refer to those studies, which, for the better part, have been conducted by bachelor and master students. The studies by Hahl et al. (2014) and Westerholm (2006) are now referred to in Section 1. Still, we find no reason why we should not study the Nordic IPO market. Our contribution to the literature is found in the method used—latent class analysis (LCA)—and not in any particular difference between the Nordic IPO market and other (small) IPO markets.

3. The extension of the time horizon from one day, to one week and to four weeks has been done several times in the IPO literature as well and did not show any spectacular additional insights. It is unclear in what way the current study would change that conclusion.

The descriptive statistics of first-day, first-week and four-week returns are *not* the main contribution of the paper. Instead, the main contribution is our use of LCA when analyzing IPO data. LCA is a statistical tool that is used extensively in research in, for example, psychology. However, to the best of our knowledge, there is no study of IPOs that uses LCA when analyzing the data. Moreover, since LCA is used on data with different time horizons, we believe it is appropriate to tabulate and discuss the properties of the data before focusing on the paper’s main contribution. Be aware that the paper has a new title to better emphasize our contribution to the literature.

4. The sample size is relatively small, about 300 IPOs, so distributing this over 4 groups renders very small subsample sizes, questioning the relevance of the results.

We believe that there is a slight misunderstanding here. LCA does not divide a sample of data into (mutually exclusive) subsamples. If we look at Table 10 (formerly Table 9), the predicted number of IPOs in each latent class is estimated.

On the one hand, if the bar is low for predicted latent class membership, then it is possible that a specific IPO may belong to more than one of the identified latent classes. For example, if we look at the first row in the table (i.e., first-day returns and probability = 0.1), the number of IPOs sum up to 238 + 160 + 8 + 8 = 414. Thus, since 414 is larger than 314, several IPOs are predicted to belong to more than one latent class.

On the other hand, if the bar is high for predicted latent class membership, then it is possible that a specific IPO does *not* belong to any of the identified latent classes. For example, if we look at the third row in the table (i.e., first-day returns and probability = 0.9), the number of IPOs sum up to 137 + 67 + 8 + 1 = 213. Hence, since 213 is smaller than 314, several IPOs are *not* predicted to belong to any of the latent classes.

In the revised paper, we clarify that LCA does not divide the sample into (mutually exclusive) subsamples. Finally, as written in the second paragraph of Section 3, four latent classes (not one, two or three classes) were identified using standard information criteria for model selection. Regarding the sample size in our study, 314 IPOs are clearly sufficient for LCA.

5. In what why is the institutional framework of Denmark, Finland, Norway and Sweden alike or different? Can we just pool all IPOs together?

All IPOs were pooled together in the previous version of the paper.

Once more, thank you for your time reading our paper.

Sincerely, Mikael Bask and Anton Läck Nätter
